# Wind Exposure and Phthisis

**Published:** 1900-10-27

**Authors:** 


### Wind Exposure and Phthisis.

At the meeting of the Royal Medical and Chirurgical Society held last Tuesday, a paper was read by Dr. W. Gordon, in which he raised the question of the influence which exposure to wind has upon the incidence of phthisis upon a locality. From a large number of observations he was able to show that the mortality from phthisis does not run with, in fact sometimes runs quite opposite to, the general death rate, and thus, by inference, that a high phthisis rate is not of necessity the result of those conditions of insanitation by which a high general death rate is produced. He then considered one by one the relation of phthisis to rainfall, to varieties of soil, and to exposure to wind; and, in regard to the latter, he referred to the conclusions arrived at by Dr. Havilland, in his work on the geographical distribution of this disease, published in 1868, in which he pointed out that exposure to the prevailing winds was associated with a high phthisis mortality. Testing this point in regard to Devonshire, Dr. Gordon said: "If the *north* wind were injurious, we could not account for the low mortality of the whole north coast. It is, no doubt, a precipitous coast, and, as such, protects the country behind it; but much of the population is distributed in valleys opening seaward, with no cliffs to protect them. The same reasoning applies to the *north-west* wind. Moreover, it is evident that the north-west wind has free access to the valleys of the northern rivers, whose course carries them through areas of low phthisis mortality. If the *north-east* wind exercised a very injurious influence it would almost reverse the actual distribution of the death-rate. The *east* wind has access to a great part of the low mortality area. The *south-east* wind has fair access to all the areas of low mortality. The *south* wind has access to all the areas of low mortality, except, perhaps, Torrington. We are therefore left with the *west* and *south-west* winds only," and he went on to show the relation of these winds to phthisis mortality, saying in conclusion: "On the whole, I think it may be probably concluded that in Devon exposure to west and south-west winds increases mortality from phthisis. It is also interesting to note how the population has avoided these winds. The villages of to-day and the hut circles of primitive ages alike are placed where west and south-west gales can least easily reach them;" and, again, "it seems . . . impossible to escape from the conclusion that, certainly for the district of St. Thomas, certainly for the district of Newton Abbot, and probably for Devonshire generally, the paramount influence which determines the higher or lower rate of phthisis mortality is the degree of exposure to or shelter from the west and south-west winds." All careful observations on this important subject are of extreme value, and much credit is due to Dr. Gordon for the trouble he has taken in collecting the statistics on which he founds his conclusions. We think, however, that it would not be well to accept too readily the suggestion that any particular wind has any particularly morbific influence, or even that the greater prevalence of phthisis in exposed situations is in any way the direct result of the winds of heaven, blow they from where they like.

Unless we are to cast aside a good deal of the progress which we have made in regard to the etiology of phthisis we must in some way correlate the observations made by Dr. Gordon with the knowledge we already possess in regard to the causative relation between well-ascertained conditions (such as overcrowding and lack of ventilation) and the prevalence of the disease. There can be no doubt that Dr. Gordon's paper bristled with weak points in that no note was taken of race, poverty, or occupation—the hardy fisherman and the sedentary lacemaker being lumped together in one common record; but accepting for the moment the general proposition that exposure to the prevailing winds increases the liability of a district to phthisis, the question still remains whether this is not the result of its encouraging habits which are well known to be provocative of consumption. Looking at the matter from this point of view anyone who has studied the habits of those who dwell on exposed hillsides is able at once to put his finger on at least one probable link between wind exposure and the prevalence of phthisis. This link is the appalling stuffiness and lack of ventilation which are so often to be found in houses exposed to prevailing winds. No one who has not had experience of open wind-swept districts knows the value placed by cottagers upon warmth, and where wood is scarce and coal has to be dragged in country carts over miles of rugged country roads, the warmth derived from shut-up rooms and closely crowded humanity is the easiest to get. Cottages on the moorland side are often picturesque enough, the tiny window panes, the creepered walls, the moss-grown roof, the deep porch often provided with inner and outer doors, are subjects that the artist loves. But the real inwardness of these appurtenances is that they keep out wind; in other words, they keep out air. The same, although perhaps in less degree, is true of windswept towns. It is not the wind that causes phthisis, but the airlessness of dwellings where every chink is stopped to keep out the fury of the gale. On the other hand it is not the absence of wind that causes a low incidence of phthisis on the lea side of great hills, but rather the fact that the inhabitants being naturally protected from great storms can leave, and do leave, their windows open without fear.

That consumption is most prevalent in wind-swept districts is an observation of great interest, but let us not be driven by it from the root-fact that it is lack of air rather than excess that leads to phthisis.

